# Supplementary material for: Subcortical serotonin 5HT2c receptor-containing neurons sex-specifically regulate binge-like alcohol consumption, social, and arousal behaviors in mice
Source: Nat Commun. 2023 Mar 31;14:1800. doi: 10.1038/s41467-023-36808-2 (PMC10066391; doi:10.1038/s41467-023-36808-2)
Supplement: Supplementary file 1 — Supplementary Information [file 41467_2023_36808_MOESM1_ESM.pdf]

Supplementary Information

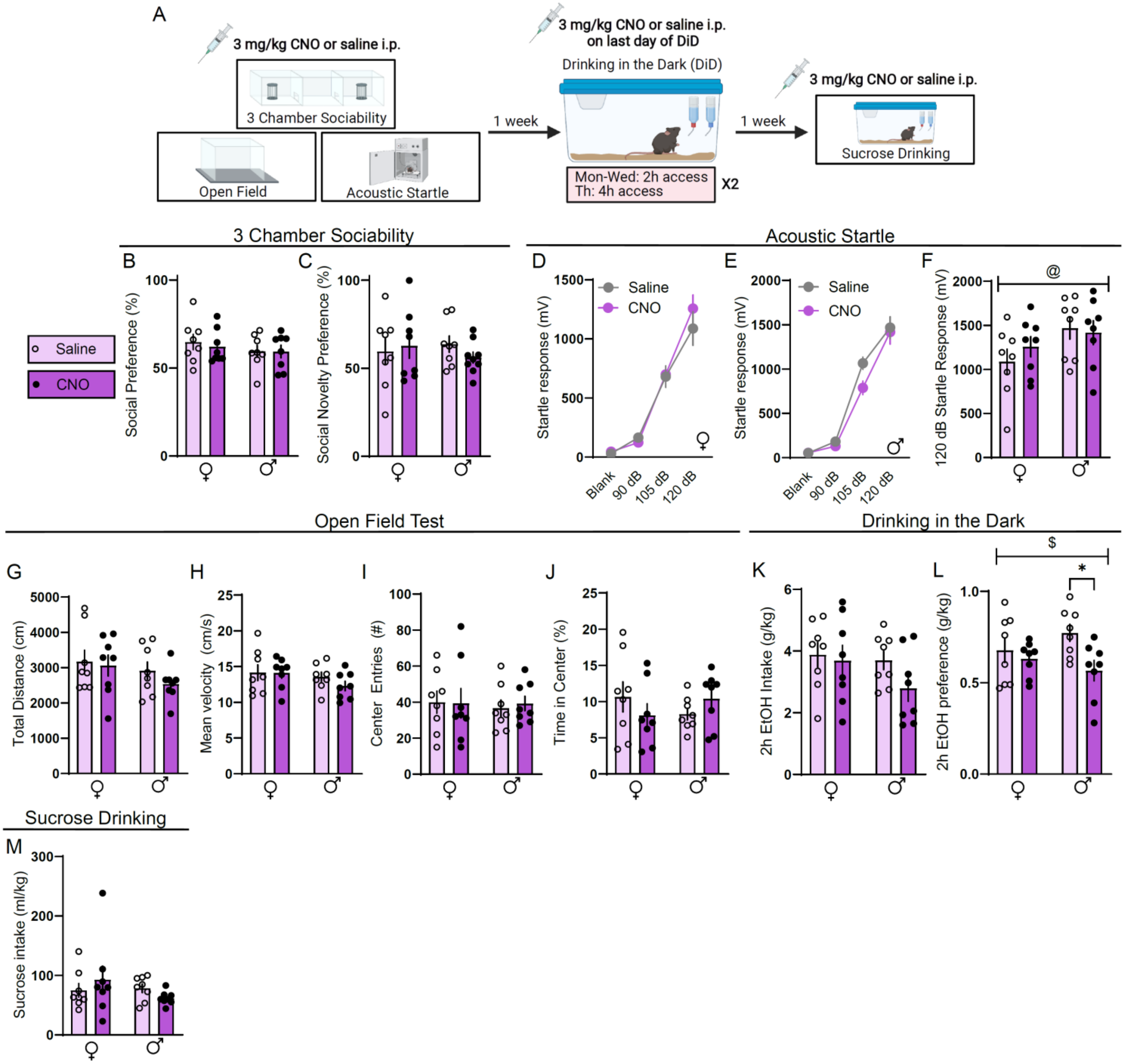

Figure S1 (accompanies Figure 3): Systemic CNO treatment effects on behavior in wild-type mice. A, Experimental timeline for CNO vs. vehicle studies. **B,** Social preference in the 3-chamber sociability test. **C,** Social novelty preference in 3-chamber sociability test. **D,** Acoustic startle behavior, females, two-way repeated measures ANOVA (10 trials/dB). **E,** Acoustic startle behavior, males (10 trials/dB), two-way repeated measures ANOVA. **F,** Acoustic startle behavior, 120 dB only (10 trials). **G,** Total distance in open field. **H,** Mean velocity in open field. **I,** Center entries in the open field. **J,** Time spent in center of open field. **K,** Alcohol intake in DiD. **L,** Alcohol preference in DiD. **M,** Sucrose consumption. For all panels, n=8 mice/group. Unless otherwise stated, statistical comparisons performed using two-way ANOVA followed by Holm-Sidak's post-hoc. All data are

presented as mean ± SEM. $ denotes effect of DiD, @ denotes effect of sex, * denotes post-hoc p<0.05. Source data are provided as a Source Data file. Created with Biorender.com.

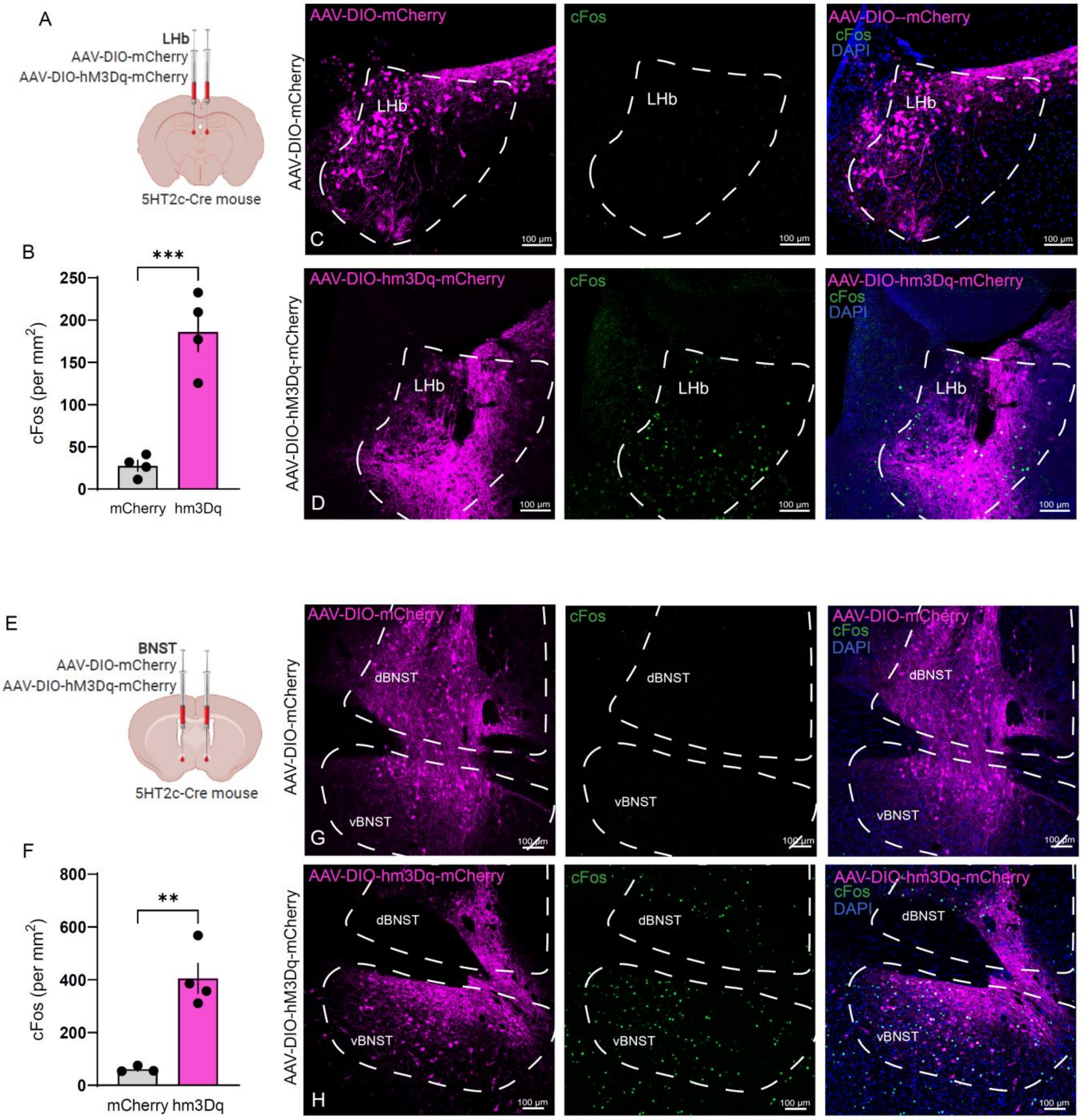

Figure S2 (accompanies Figure 3): *In-vivo* validation of chemogenetic activation approach. A, Surgical schematic for chemogenetic activation of LHb_{5HT2c} neurons. **B,** Quantification of c-Fos following systemic CNO administration. **C,** Representative viral infection (left), c-Fos (middle), and composite (right) for AAV-DIO-mCherry LHb condition. Similar images were obtained in n=4 mice. **D,** Representative viral infection (left), c-Fos (middle), and composite (right) for AAV-DIO-hm3Dq-mCherry LHb condition. Similar images were obtained in n=4 mice. **E,** Surgical schematic for chemogenetic activation of $BNST_{5HT2c}$ neurons. **F,** Quantificaiton of c-Fos following systemic CNO administration. **G,** Representative viral infection (left), c-Fos (middle), and composite (right) for AAV-DIO-mCherry BNST condition. Similar images were obtained in n=3 mice. **H,** Representative viral

infection (left), c-Fos (middle), and composite (right) for AAV-DIO-hm3Dq-mCherry BNST condition. Similar images were obtained in n=4 mice. For LHb, mCherry n=3 mice/2 slices per mouse, hM3Dq n=4 mice/2 slices per mouse. For BNST, mCherry n=3 mice/2 slices per mouse, hM3Dq n=4 mice/2 slices per mouse. Statistical comparisons were performed using two-tailed *Student's* unpaired t-tests. All data presented as mean $\pm$ SEM. **p>0.01, ***p<0.001. Source data are provided as a Source Data file. Created with Biorender.com.

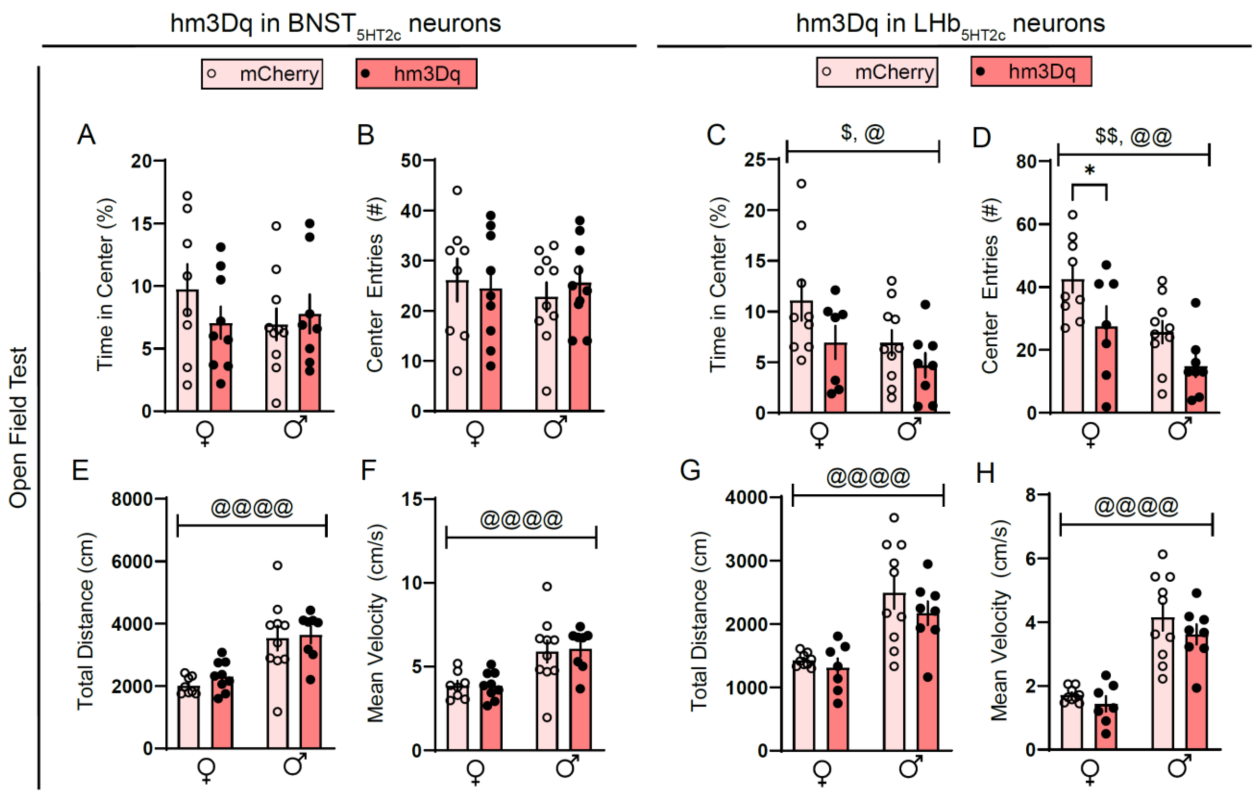

Figure S3 (accompanies Figure 3): Effects of chemogenetic activation of LHb_{5HT2c} or $BNST_{5HT2c}$ neurons on open field behavior. A, Time in center of open field for $BNST_{5HT2c}$ activation. **B,** Center entries in the open field $BNST_{5HT2c}$ activation. **C,** Time in center of open field for LHb_{5HT2c} activation. **D,** Entries in center of open field for LHb_{5HT2c} activation. **E,** Total distance in open field for $BNST_{5HT2c}$ activation. **F,** Mean velocity in open field for $BNST_{5HT2c}$ activation. **G,** Total distance in open field for LHb_{5HT2c} activation. **H,** Mean velocity in open field for LHb_{5HT2c} activation. BNST, Females: mcherry n=8 mice, hM3Dq n=9 mice; Males: mCherry n=10 mice, hM3Dq n=8 mice. LHb, Females: mCherry n=9 mice, hM3Dq n=7 mice; Males: mCherry n=9 mice, hM3Dq n=8 mice. Statistical comparisons were performed using two-way ANOVA followed by Holm-Sidak's post-hoc. All data presented as mean $\pm$ SEM. \$ denotes effect of virus (\$p<0.05, \$\$p<0.01), @ denotes effect of sex (@ p<0.05, @@p<0.01, @@@p<0.001, @@@@p<0.0001), and *denote post-hoc p<0.05. Source data are provided as a Source Data file.

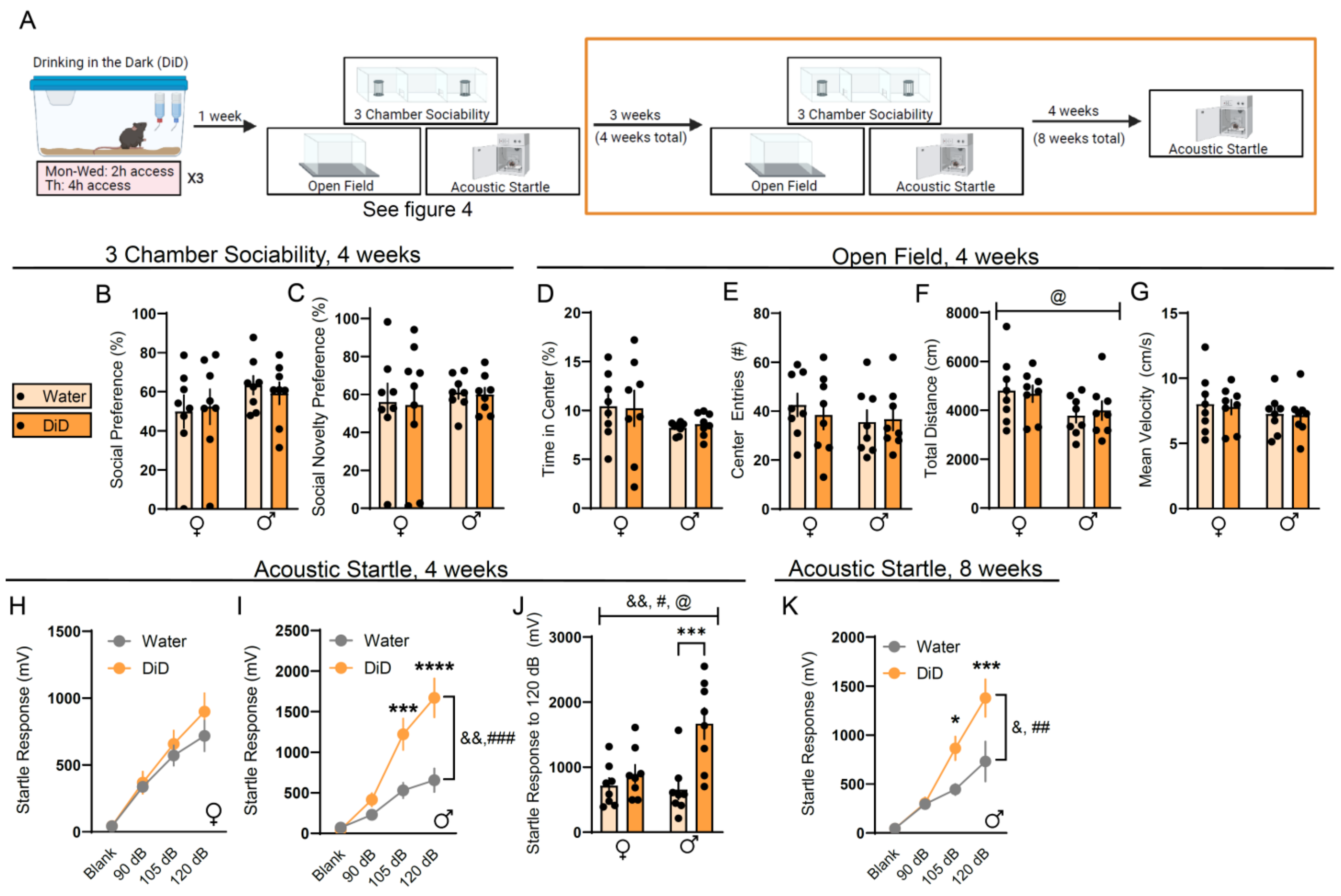

Figure S4 (accompanies Figure 4): DiD induces long-lasting changes in male acoustic startle behavior. **A,** Experimental timeline for investigating effects of DiD on affective behaviors. This figure is specifically showing behaviors at the time points in the orange box. **B,** Social preference in 3-chamber sociability test. **C,** Social novelty preference in the 3-chamber sociability test. **D,** Time in center of open field. **E,** Center entries in open field. **F,** Total distance in open field. **G,** Mean velocity in an open field. **H,** Acoustic startle behavior at four weeks, females, two-way repeated measures ANOVA. **I,** Acoustic startle behavior at 4 weeks, males, two-way repeated measures ANOVA followed by Holm-Sidak's post-hoc. **J,** Acoustic startle behavior at four weeks, 120 dB only. **K,** Acoustic startle behavior at 8 weeks, males, two-way repeated measures ANOVA followed by Holm-Sidak's post-hoc. Females: water n=8 mice, DiD n=8 mice; Males: water n=8 mice, DiD n=8 mice. Unless otherwise stated, statistical comparisons were performed using two-way ANOVAs followed by Holm-Sidak's post-hoc. All data are represented as mean ± SEM. & denotes effects of DiD, @ denotes effect of sex, # denotes interaction, *denote post-hoc effects. Source data are provided as a Source Data file. Created with Biorender.com.

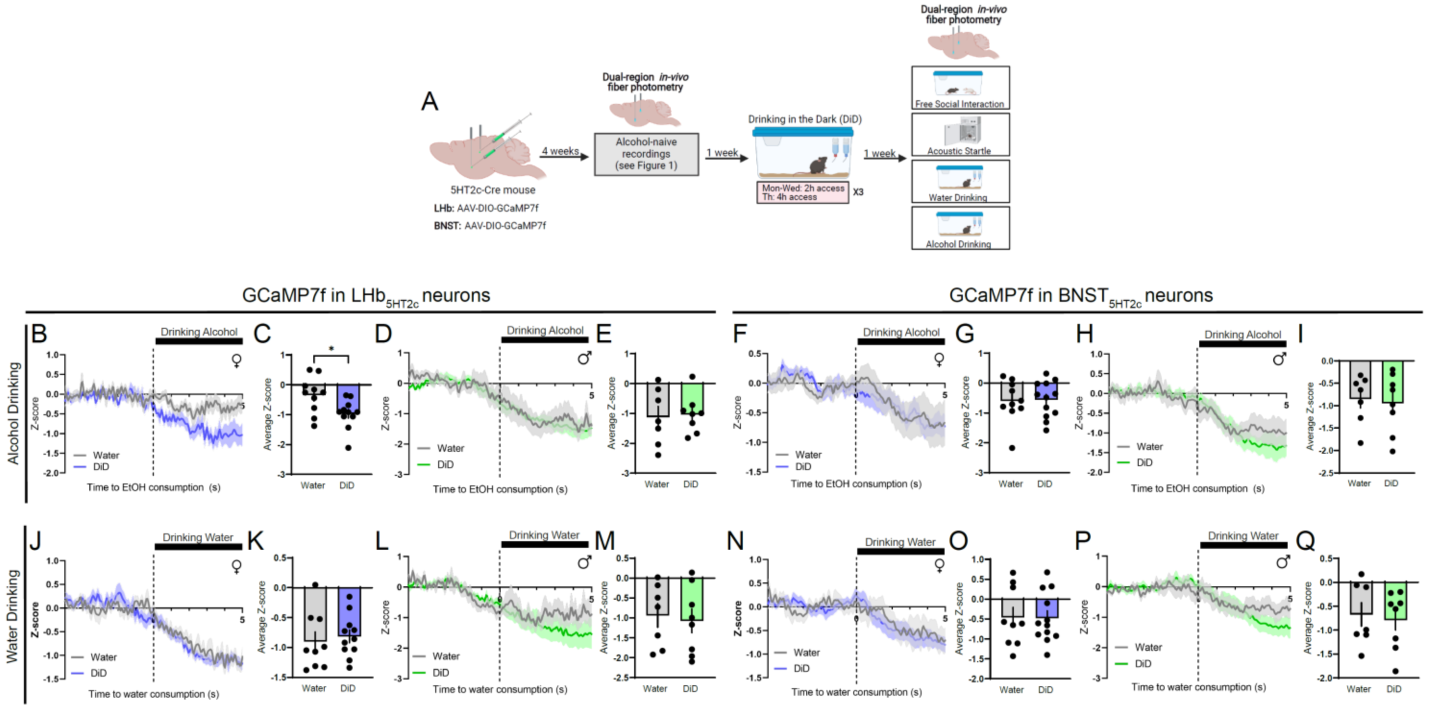

Figure S5 (accompanies Figure 6): DiD alters calcium (GCaMP) responses of LHb_{5HT2c} neurons in females. A, Surgical schematic and experimental timeline for LHb_{5HT2c} and $BNST_{5HT2c}$ GCaMP recordings. **B,** Peri-event plot of LHb_{5HT2c} GCaMP activity during alcohol drinking, females (water n=10 mice/1-4 bouts; DiD n=11 mice/1-4 bouts). **C,** Average z-score of LHb_{5HT2c} GCaMP activity for 0-5s post bout start, females (water n=10 mice/1-4 bouts; DiD n=11 mice/1-4 bouts). **D,** Peri-event plot of LHb_{5HT2c} GCaMP activity during alcohol drinking, males (water n=8 mice/1-4 bouts, DID=10 mice/1-4 bouts). **E,** Average z-score of LHb_{5HT2c} GCaMP signal for 0-5s post bout start, males (water n=8 mice/1-4 bouts, DID=10 mice/1-4 bouts). **F,** Peri-event plot of $BNST_{5HT2c}$ GCaMP activity during alcohol drinking, females (water n=10 mice/1-4 bouts; DiD n=12 mice/1-4 bouts). **G,** Average z-score of $BNST_{5HT2c}$ GCaMP signal for 0-5s post bout start, females (water n=10 mice/1-4 bouts; DiD n=12 mice/1-4 bouts). **H,** Peri-event plot of $BNST_{5HT2c}$ GCaMP signal during alcohol drinking, males (water n=7 mice/1-4 bouts; DiD n=8 mice/1-4 bouts). **I,** Average z-score of $BNST_{5HT2c}$ GCaMP signal for 0-5s post bout start, males (water n=7 mice/1-4 bouts; DiD n=8 mice/1-4 bouts). **J,** Peri-event plot of LHb_{5HT2c} GCaMP activity during water drinking, females (water n=9 mice/1-4 bouts; DiD n=11 mice/1-4 bouts). **K,** Average z-score of LHb_{5HT2c} signal for 0-5s post bout start, females (water n=9 mice/1-4 bouts; DiD n=11 mice/1-4 bouts). **L,** Peri-event plot of LHb_{5HT2c} GCaMP activity during water drinking, males (water n=7 mice/1-4 bouts; DiD n=8 mice/1-4 bouts). **M,** Average z-score of LHb_{5HT2c} GCaMP signal for 0-5s post bout start, males (water n=7 mice/1-4 bouts; DiD n=8 mice/1-4 bouts). **N,** Peri-event plot of $BNST_{5HT2c}$ GCaMP activity during water drinking, females (water n=9 mice/1-4 bouts; DiD n=12 mice/1-4 bouts). **O,** Average z-score of $BNST_{5HT2c}$ GCaMP signal for 0-5s post bout start (water n=9 mice/1-4 bouts; DiD n=12 mice/1-4 bouts). **P,** Peri-event plot of $BNST_{5HT2c}$ GCaMP activity during water drinking, males (water n=7 mice/1-4 bouts; DiD n=8 mice/1-4 bouts). **Q,** Average z-score of $BNST_{5HT2c}$ GCaMP signal for 0-5s post bout start. Statistical comparisons were performed using two-tailed *Student's* unpaired t-tests. All data are presented as mean $\pm$ SEM. *p<0.05. Source data are provided as a Source Data file. Created with Biorender.com.

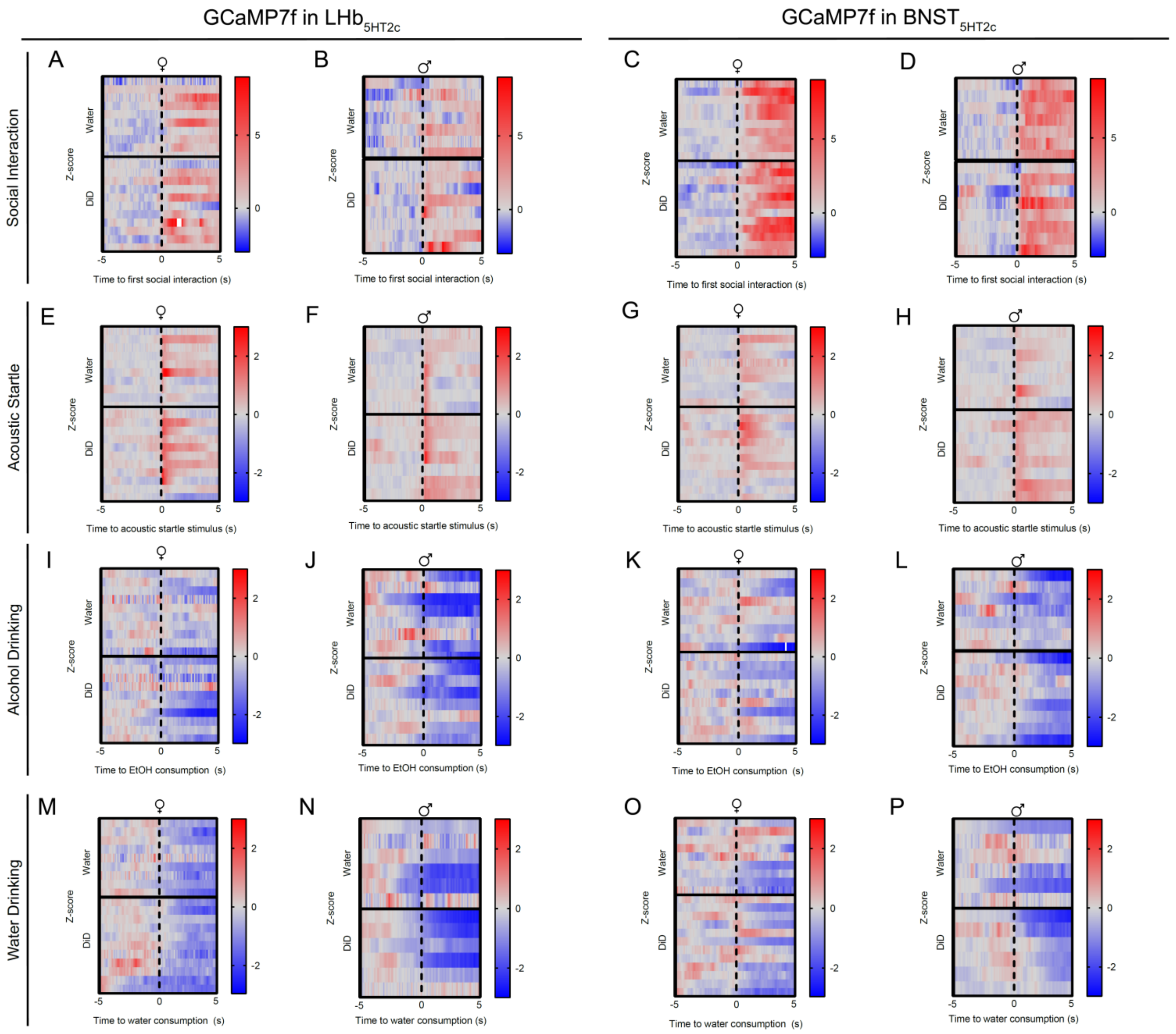

Figure S6 (accompanies Figure 7): Individual animal responses for GCaMP7f experiments. A, Free social interaction, LHb_{5HT2c} females. **B,** Free social interaction, LHb_{5HT2c} males. **C,** Free social interaction, $BNST_{5HT2c}$ females. **D,** Free social interaction, $BNST_{5HT2c}$ males. **E,** Acoustic startle, LHb_{5HT2c} females. **F,** Acoustic startle, LHb_{5HT2c} males. **G,** Acoustic startle, $BNST_{5HT2c}$ females. **H,** Acoustic startle, $BNST_{5HT2c}$ males. **I,** Alcohol drinking, LHb_{5HT2c} females. **J,** Alcohol drinking, LHb_{5HT2c} males. **K,** Alcohol drinking, $BNST_{5HT2c}$ females. **L,** Alcohol drinking, $BNST_{5HT2c}$ males. **M,** Water drinking, LHb_{5HT2c} females. **N,** Water drinking, LHb_{5HT2c} males. **O,** Water drinking, $BNST_{5HT2c}$ males. **P,** Water drinking, $BNST_{5HT2c}$ males. Each horizontal line in the heatmap corresponds to the average of all trials for a single animal. Source data are provided as a Source Data file.

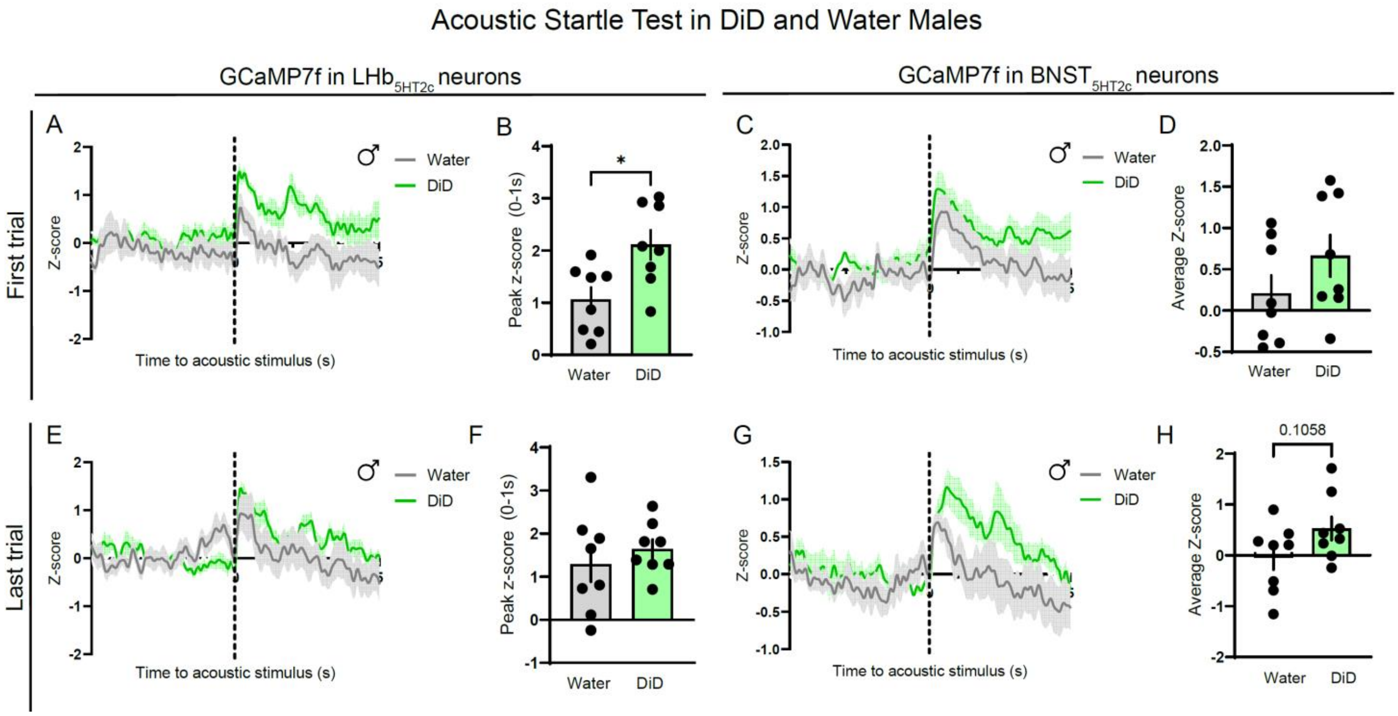

Figure S7 (accompanies Figure 7): 5HT2c-containing neurons alter their responses to stimuli over time. **A,** Peri-event plot of first startle trial, LHb_{5HT2c} GCaMP DiD vs. Water males. **B,** Peak z-score of first startle trial for 0-1s post startle stimulus, LHb_{5HT2c} GCaMP DiD vs. Water males. **C,** Peri-event plot of first startle trial, $BNST_{5HT2c}$ GCaMP DiD vs. Water males. **D,** Average z-score of first startle trial, $BNST_{5HT2c}$ GCaMP DiD vs. Water males for 0-5s post startle stimulus. **E,** Peri-event plot of last startle trial, LHb_{5HT2c} GCaMP DiD vs. Water males. **F,** Peak z-score of last startle trial, LHb_{5HT2c} GCaMP DiD vs. Water males for 0-1s post startle stimulus. **G,** Peri-event plot of last startle trial, $BNST_{5HT2c}$ GCaMP DiD vs. Water males. **H,** Average z-score of last startle trial, $BNST_{5HT2c}$ GCaMP DiD vs. Water males for 0-5s post startle stimulus. For all panels, water n=8 mice/1 trial, DiD n=8 mice/1 trial. Statisitcal comparisons were performed using two-tailed *Student's* unpaired t-tests. All data presented as mean $\pm$ SEM. *$p<0.05$. Source data are provided as a Source Data file.

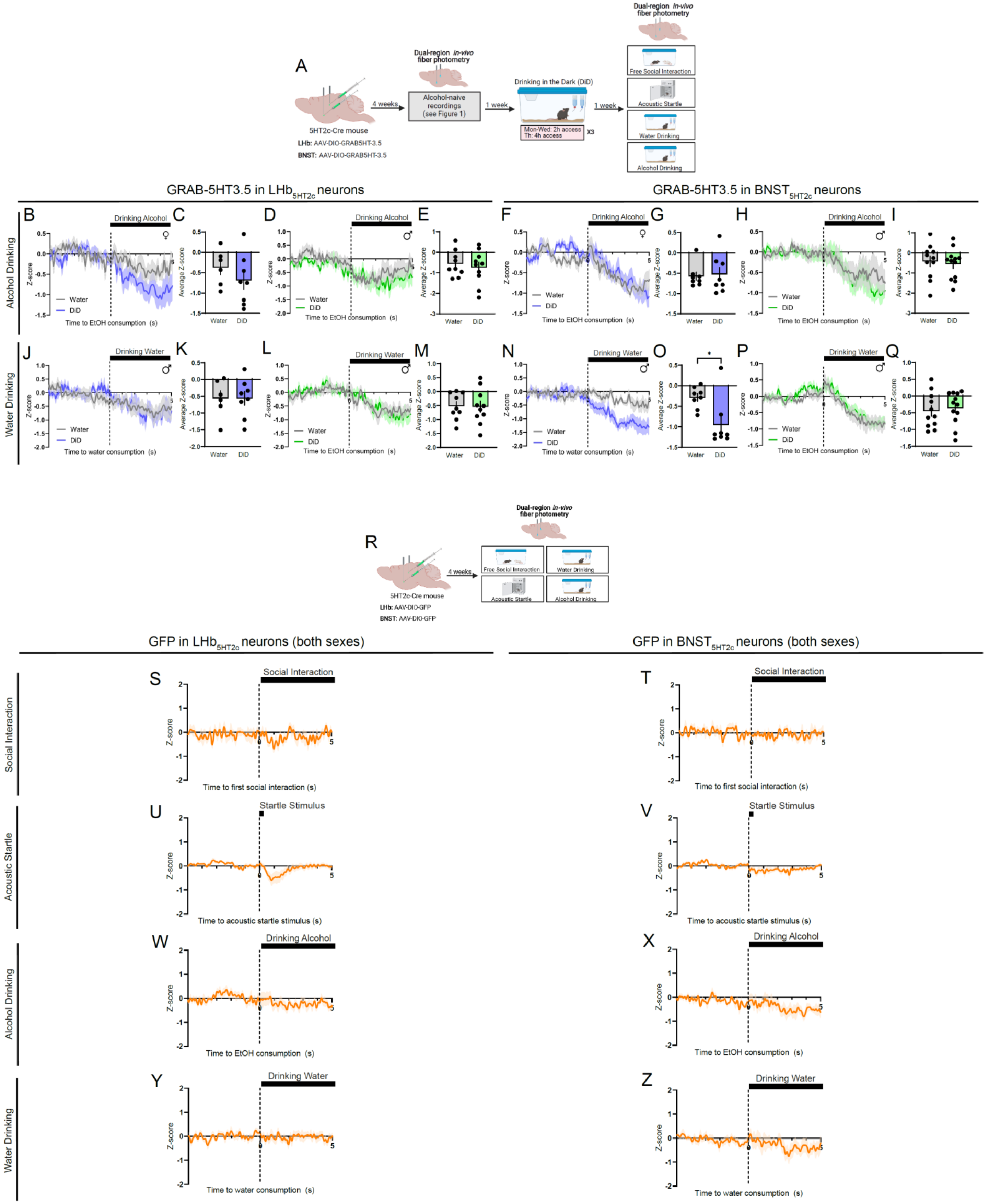
GRAB-5HT3.5 in LHb5HT2c neurons
GRAB-5HT3.5 in BNST5HT2c neurons
Alcohol Drinking
Water Drinking
Drinking Alcohol
Drinking Water
Time to EtOH consumption (s)
Time to water consumption (s)
Water
DiD
GFP in LHb5HT2c neurons (both sexes)
GFP in BNST5HT2c neurons (both sexes)
Social Interaction
Acoustic Startle
Startle Stimulus
Time to first social interaction (s)
Time to acoustic startle stimulus (s)
5HT2c-Cre mouse
LHb: AAV-DIO-GRAB5HT-3.5
BNST: AAV-DIO-GRAB5HT-3.5
LHb: AAV-DIO-GFP
BNST: AAV-DIO-GFP
Drinking in the Dark (DiD)
Dual-region in-vivo fiber photometry

Figure S8 (accompanies Figure 8): DiD alters 5-HT release onto $BNST_{5HT2c}$ neurons in females during water drinking; GFP motion controls. A, Surgical schematic and experimental timeline for LHb_{5HT2c} and $BNST_{5HT2c}$ GRAB-5HT recordings. **B,** Peri-event plot of LHb_{5HT2c} GRAB-5HT activity during alcohol drinking, females (water n=6 mice/1-4 bouts; DiD n=8 mice/1-4 bouts). **C,** Average z-score of LHb_{5HT2c} GRAB-5HT activity for 0-5s post bout start, females (water n=6 mice/1-4 bouts; DiD n=8 mice/1-4 bouts). **D,** Peri-event plot of LHb_{5HT2c} GRAB-5HT activity during alcohol drinking, males (water n=9 mice/1-4 bouts; DiD n=10 mice/1-4 bouts). **E,** Average z-score of LHb_{5HT2c} GRAB-5HT signal for 0-5s post bout start, males (water n=9 mice/1-4 bouts; DiD n=10 mice/1-4 bouts). **F,** Peri-event plot of $BNST_{5HT2c}$ GRAB-5HT activity during alcohol drinking, females (water n=8 mice/1-4 bouts; DiD n=8 mice/1-4 bouts). **G,** Average z-score of $BNST_{5HT2c}$ GRAB-5HT signal for 0-5s post bout start, females (water n=8 mice/1-4 bouts; DiD n=8 mice/1-4 bouts). **H,** Peri-event plot of $BNST_{5HT2c}$ GRAB-5HT signal during alcohol drinking, males (water n=11 mice/1-4 bouts; DiD n=12 mice/1-4 bouts). **I,** Average z-score of $BNST_{5HT2c}$ GRAB-5HT signal for 0-5s post bout start, males (water n=11 mice/1-4 bouts; DiD n=12 mice/1-4 bouts). **J,** Peri-event plot of LHb_{5HT2c} GRAB-5HT activity during water drinking, females (water n=6 mice/1-4 bouts; DiD n=8 mice/1-4 bouts). **K,** Average z-score of LHb_{5HT2c} signal for 0-5s post bout start, females (water n=6 mice/1-4 bouts; DiD n=8 mice/1-4 bouts). **L,** Peri-event plot of LHb_{5HT2c} GRAB-5HT activity during water drinking, males (water n=9 mice/1-4 bouts; DID n=10 mice/1-4 bouts). **M,** Average z-score of LHb_{5HT2c} GRAB-5HT signal for 0-5s post bout start, males (water n=9 mice/1-4 bouts; DID n=10 mice/1-4 bouts). **N,** Peri-event plot of $BNST_{5HT2c}$ GRAB-5HT activity during water drinking, females (water n=8 mice/1-4 bouts; DiD n=8 mice/1-4 bouts). **O,** Average z-score of $BNST_{5HT2c}$ GRAB-5HT signal for 0-5s post bout start (water n=8 mice/1-4 bouts; DiD n=8 mice/1-4 bouts). **P,** Peri-event plot of $BNST_{5HT2c}$ GRAB-5HT activity during water drinking, males (water n=11 mice/1-4 bouts; DiD n=12 mice/1-4 bouts). **Q,** Average z-score of $BNST_{5HT2c}$ GRAB-5HT signal for 0-5s post bout start (water n=11 mice/1-4 bouts; DiD n=12 mice/1-4 bouts). **R,** Surgical schematic and experimental timeline for LHb_{5HT2c} and $BNST_{5HT2c}$ GFP control photometry experiments. **S,** Peri-event plot of LHb_{5HT2c} GFP signal during free social interaction (n=9 mice, 1 trial/mouse). **T,** Peri-event plot of $BNST_{5HT2c}$ GFP signal during free social interaction (n=9 mice, 1 trial/mouse). **U,** Peri-event plot of LHb_{5HT2c} GFP signal during the acoustic startle test (n=9 mice, average of 10 trials/mouse). **V,** Peri-event plot of $BNST_{5HT2c}$ GFP signal during the acoustic startle test (n=9 mice, average of 10 trials/mouse). **W,** Peri-event plot of LHb_{5HT2c} GFP signal during voluntary alcohol drinking (n=9 mice, average of 1-3 bouts/mouse). **X,** Peri-event plot of $BNST_{5HT2c}$ GFP signal during voluntary alcohol consumption (n=9 mice, average of 1-4 bouts/mouse). **Y,** Peri-event plot of LHb_{5HT2c} GFP signal during voluntary water drinking (n=9 mice, average of 1-4 bouts/mouse). **Z,** Peri-event plot of $BNST_{5HT2c}$ GFP signal during voluntary water drinking (n=9 mice, average of 1-4 bouts/mouse). Statistical comparisons were performed using two-tailed *Student's* unpaired t-tests. All data are presented as mean $\pm$ SEM. *$p<0.05$. Source data are provided as a Source Data file. Created with Biorender.com.

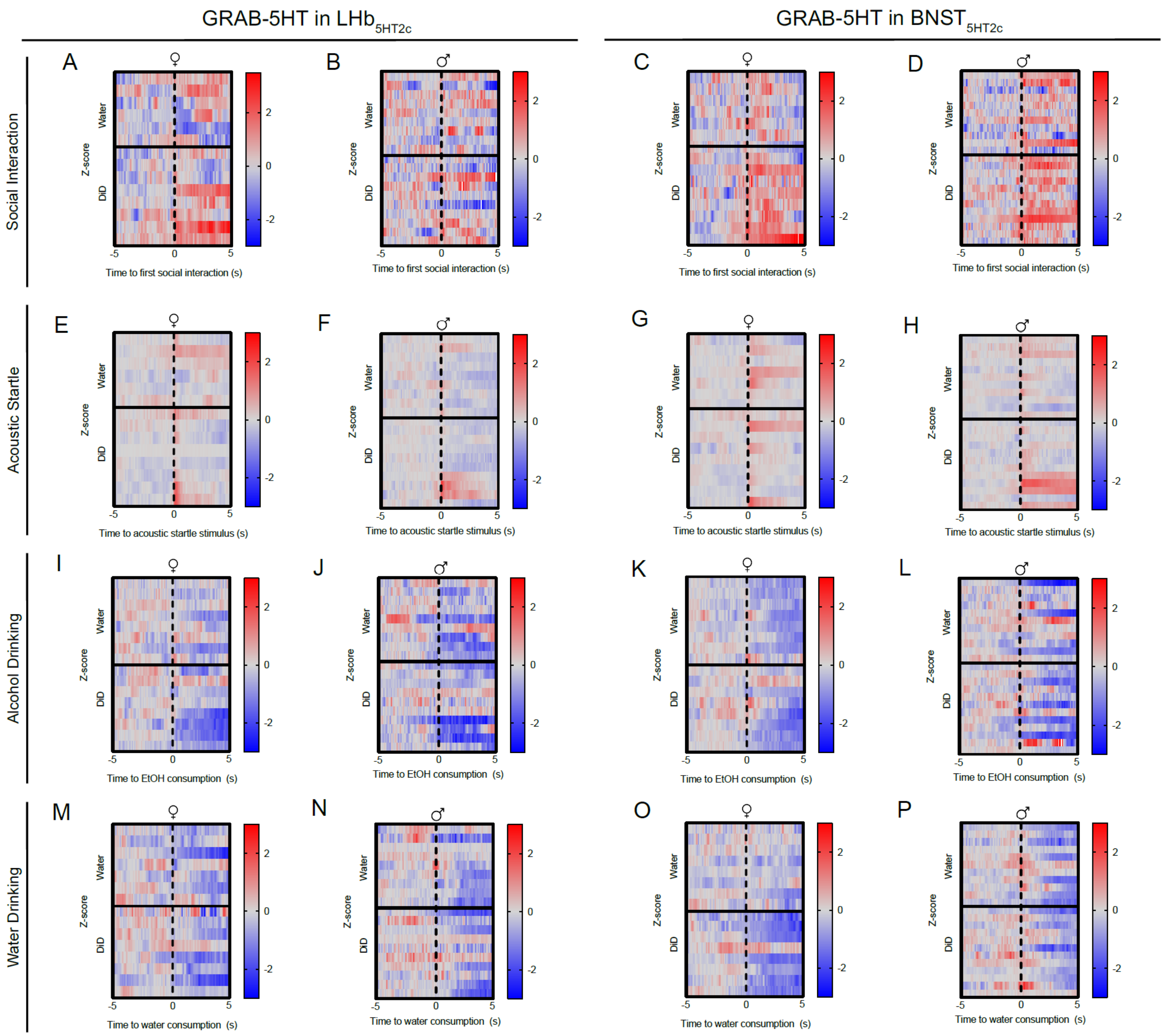

Figure S9 (accompanies Figure 8): Individual animal responses for GRAB-5HT experiments. A, Free social interaction, LHb_{5HT2c} females. **B,** Free social interaction, LHb_{5HT2c} males. **C,** Free social interaction, $BNST_{5HT2c}$ females. **D,** Free social interaction, $BNST_{5HT2c}$ males. **E,** Acoustic startle, LHb_{5HT2c} females. **F,** Acoustic startle, LHb_{5HT2c} males. **G,** Acoustic startle, $BNST_{5HT2c}$ females. **H,** Acoustic startle, $BNST_{5HT2c}$ males. **I,** Alcohol drinking, LHb_{5HT2c} females. **J,** Alcohol drinking, LHb_{5HT2c} males. **K,** Alcohol drinking, $BNST_{5HT2c}$ females. **L,** Alcohol drinking, $BNST_{5HT2c}$ males. **M,** Water drinking, LHb_{5HT2c} females. **N,** Water drinking, LHb_{5HT2c} males. **O,** Water drinking, $BNST_{5HT2c}$ males. **P,** Water drinking, $BNST_{5HT2c}$ males. Each horizontal line in the heatmap corresponds to the average of all trials for a single animal.

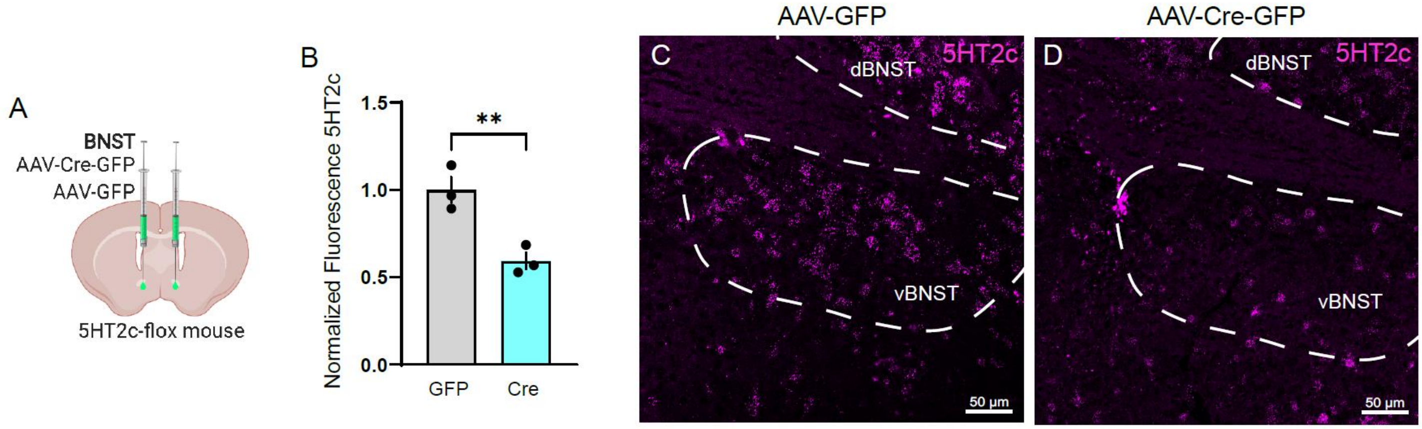

Figure S10 (accompanies Figure 9): Validation of $5HT_{2c}$ knockdown in our $5HT_{2c}^{lox/lox}$ mouse. A, Surgical schematic for $5HT_{2c}$ knockdown. **B,** Quantification of 5HT2c fluorescence. **C,** Representative 5HT2c fluorescence in AAV-GFP condition. Similar images were obtained in n=3 mice. **D,** Representative 5HT2c fluorescence in AAV-Cre-GFP condition. Similar images were obtained in n=3 mice. GFP: n=3 mice/2 slices per mouse; Cre: n=3 mice/2 slices per mouse. Statistical comparisons were performed using two-tailed *Student's* unpaired t-test. All data presented as mean ± SEM. **p<0.01. Source data are provided as a Source Data file. Created with Biorender.com.

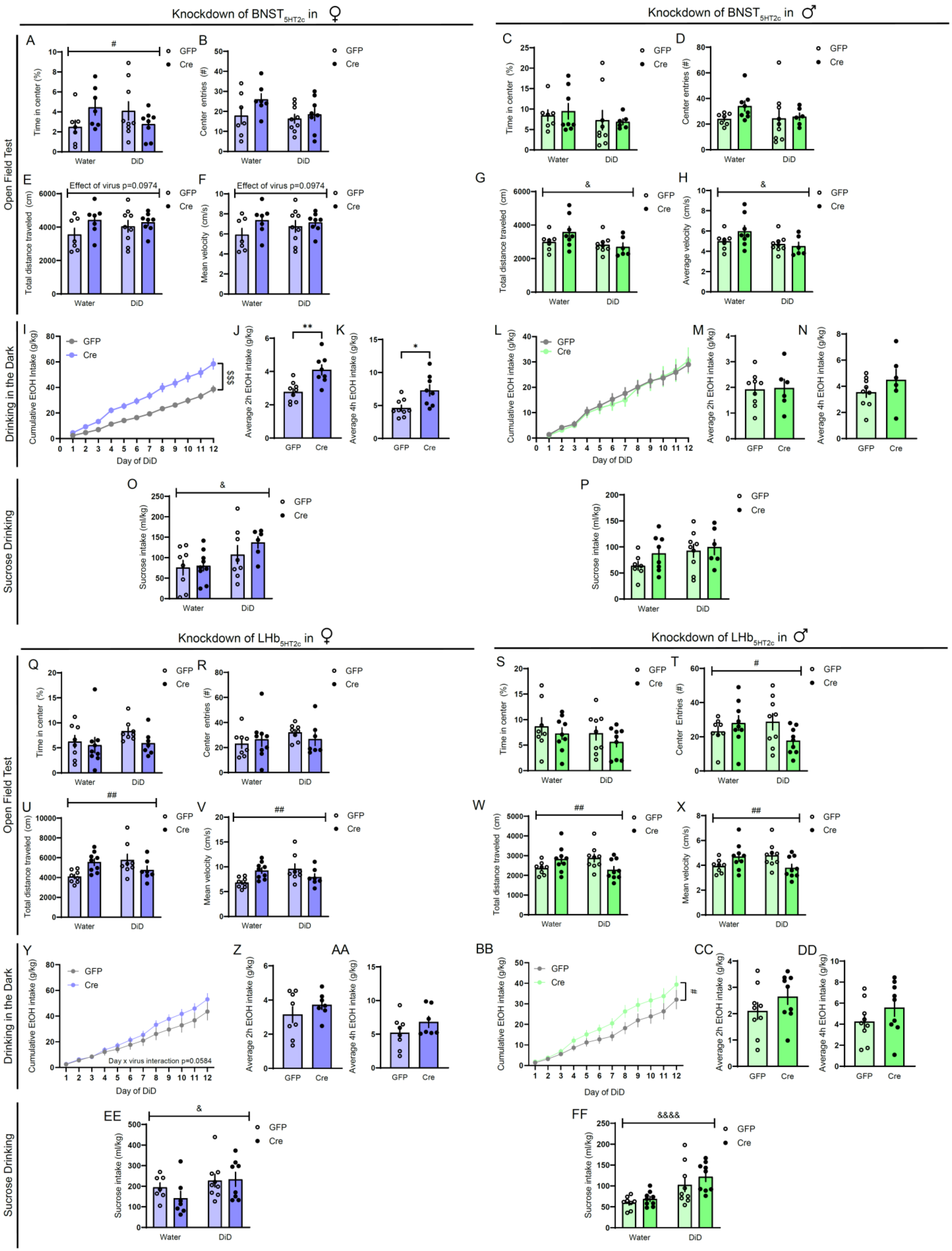
Knockdown of BNST5HT2c in ♀
Knockdown of BNST5HT2c in ♂
Open Field Test
A
#
Time in center (%)
GFP
Cre
Water
DiD
B
Center entries (#)
C
Time in center (%)
D
Center entries (#)
E
Effect of virus p=0.0974
Total distance traveled (cm)
F
Effect of virus p=0.0974
Mean velocity (cm/s)
G
&
Total distance traveled (cm)
H
&
Average velocity (cm/s)
Drinking in the Dark
I
Cumulative EtOH intake (g/kg)
Day of DiD
$$$
J
**
Average 2h EtOH intake (g/kg)
K
*
Average 4h EtOH intake (g/kg)
L
M
N
Sucrose Drinking
O
&
Sucrose intake (ml/kg)
P
Knockdown of LHb5HT2c in ♀
Knockdown of LHb5HT2c in ♂
Q
R
S
T
#
Center Entries (#)
U
##
V
##
W
##
X
##
Y
Day x virus interaction p=0.0584
Z
AA
BB
#
CC
DD
EE
&
FF
&&&&

Figure S11 (accompanies Figure 9): Effects of $5HT_{2c}$ knockdown on open field behavior, DiD, and sucrose consumption. A, Time in center of open field, BNST females. **B,** Center entries in open field, BNST females. **C,** Time in center of open field, BNST males. **D,** Center entries in open field, BNST males. **E,** Total distance in open field, BNST females. **F,** Mean velocity in open field, BNST females. **G,** Total distance in open field, BNST males. **H,** Mean velocity in open field, BNST males. **I,** Cumulative alcohol consumption in DiD, BNST females, two-way repeated measures ANOVA. **J,** Average 2h alcohol intake in DiD, BNST females. **K,** Average 4h alcohol intake in DiD, BNST females. **L,** Cumulative alcohol consumption in DiD, BNST males, two-way repeated measures ANOVA. **M,** Average 2h alcohol intake in DiD, BNST males. **N,** Average 4h alcohol intake in DiD, BNST males. **O,** Sucrose intake, BNST females. **P,** Sucrose intake, BNST males. **Q,** Time in center of open field, LHb females. **R,** Center entries in open field, LHb females. **S,** Time in center of open field, LHb males. **T,** Center entries in open field, LHb males. **U,** Total distance in open field, LHb females. **V,** Mean velocity in open field, LHb females. **W,** Total distance in open field, LHb males. **X,** Mean velocity in open field, LHb males. **Y,** Cumulative alcohol consumption in DiD, LHb females, two-way repeated measures ANOVA. **Z,** Average 2h alcohol intake in DiD, LHb females. **AA,** Average 4h alcohol intake in DiD, LHb females. **BB,** Cumulative alcohol consumption in DiD, LHb males, two-way repeated measures ANOVA. **CC,** Average 2h alcohol intake in DiD, males. **DD,** Average 4h alcohol intake in DiD, LHb males. **EE,** Sucrose intake, females. **FF,** Sucrose intake, males. BNST females, GFP: water n=7 mice, DiD n=9 mice; DiD water n=7 mice, DiD n=8 mice. BNST males, GFP: water n=7 mice, DiD n=9 mice; Cre: water n=8 mice, DiD n=6 mice. LHb females, GFP: water n=8 mice, DiD n=8 mice; Cre: water n=9 mice, DiD n=6 mice. LHb males, GFP: water n=8 mice, DiD n=9 mice; Cre: water n=9 mice, DiD n=9 mice. Unless otherwise stated, statistical comparisons between two or more groups were performed using two-way ANOVA followed by Holm-Sidak's post-hoc. Statistical comparisons between two groups were performed using two-tailed *Student's* unpaired t-tests. All data presented as mean $\pm$ SEM. & denotes effect of DiD, $ denotes effect of virus ($p<0.05, $$p<0.01, $$$p<0.0001), # denotes interaction (day x virus or DiD x virus) (#p<0.05, ##p<0.01), and * denotes post-hoc p<0.05, ** p<0.01. Source data are provided as a Source Data file.

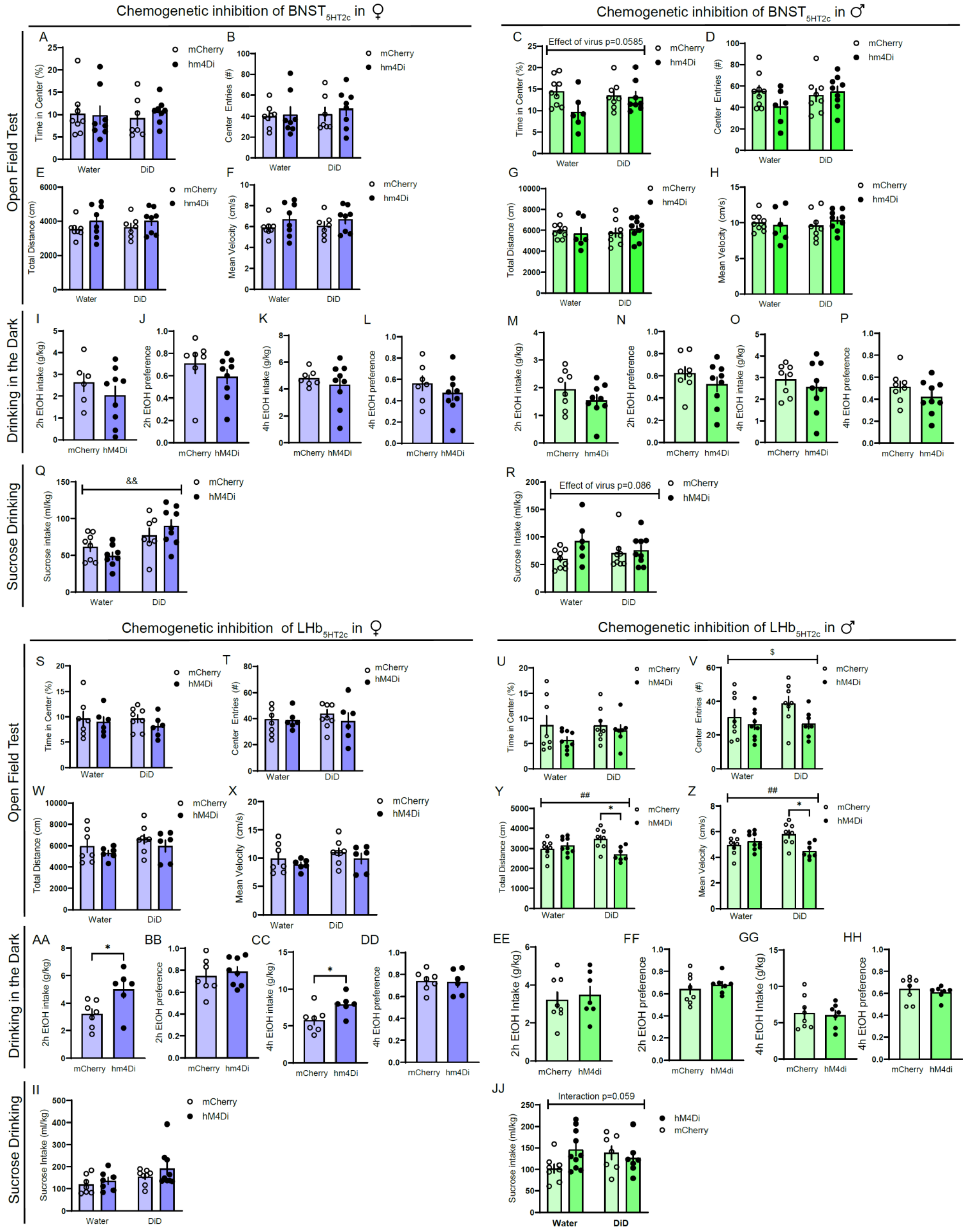
Chemogenetic inhibition of BNST5HT2c in ♀
Chemogenetic inhibition of BNST5HT2c in ♂
Open Field Test
Drinking in the Dark
Sucrose Drinking
A
Time in Center (%)
Water
DiD
mCherry
hm4Di
B
Center Entries (#)
C
Effect of virus p=0.0585
D
E
Total Distance (cm)
F
Mean Velocity (cm/s)
G
H
I
2h EtOH intake (g/kg)
J
2h EtOH preference
K
4h EtOH intake (g/kg)
L
4h EtOH preference
M
N
O
P
mCherry
hM4Di
Q
&&
Sucrose intake (ml/kg)
R
Effect of virus p=0.086
Chemogenetic inhibition of LHb5HT2c in ♀
Chemogenetic inhibition of LHb5HT2c in ♂
S
T
U
V
$
W
X
Y
##
*
Z
AA
BB
CC
DD
EE
FF
GG
HH
2h EtOH Intake (g/kg)
4h EtOH Intake (g/kg)
hM4di
II
Sucrose Intake (ml/kg)
JJ
Interaction p=0.059

Figure S12 (accompanies Figure 10): Effects of chemogenetic inhibition of LHb_{5HT2c} and $BNST_{5H2c}$ on open field behavior, DiD, and sucrose consumption. A, Time in center of open field (%), BNST females. **B,** Center entries in open field, BNST females. **C,** Time in center of open field (%), BNST males. **D,** Center entries in open field, BNST males. **E,** Total distance traveled in open field, BNST females. **F,** Mean velocity in open field, BNST females. **G,** Total distance traveled in open field, BNST males. **H,** Mean velocity in open field, BNST males. **I,** 2 hr alcohol intake in DiD, BNST females. **J,** 2 hr alcohol preference in DiD, BNST females. **K,** 4h alcohol intake in DiD, BNST females. **L,** 4h alcohol preference in DiD, BNST females. **M,** 2h alcohol intake in DiD, BNST males. **N,** 2h alcohol preference in DiD, BNST males. **O,** 4h alcohol intake in DiD, BNST males. **P,** 4h alcohol preference in DiD, BNST males. **Q,** 4h sucrose intake, BNST females. **R,** 4h sucrose intake, BNST males. **S,** Time in center of open field (%), LHb females. **T,** Center entries to open field, LHb females. **U,** Time in center of open field (%), LHb males. **V,** Center entries to open field, LHb males. **W,** Total distance traveled in open field, LHb females. **X,** Mean velocity in open field, LHb females. **Y,** Total distance traveled in open field, LHb males. **Z,** Mean velocity in open field, LHb males. **AA,** 2h alcohol intake in DiD, LHb females. **BB,** 2h alcohol preference in DiD, LHb females. **CC,** 4h alcohol intake in DiD, LHb females. **DD,** 4h alcohol preference in DiD, LHb females. **EE,** 2h alcohol intake in DiD, LHb males. **FF,** 2h alcohol preference in DiD, LHb males. **GG,** 4h alcohol intake in DiD, LHb males. **HH,** 4h alcohol preference in DiD, LHb males. **II,** 4h sucrose intake, LHb females. **JJ,** 4h sucrose intake, LHb males. LHb females, mCherry: water n=7 mice, DiD n=8 mice; hM4Di: water n=6 mice, DiD n=6 mice. LHb males, mCherry: water n=8 mice, DiD n=8 mice; hM4Di: water n=9 mice, DiD n=7 mice. BNST females, mCherry: water n=8 mice, DiD n=7 mice; hM4Di: water n=8 mice, DiD n=9 mice. BNST males, mCherry: water n=9 mice, DID n=8 mice; hM4Di water n=6 mice, DiD n=9 mice. Statistical comparisons between two or more groups were performed using two-way ANOVA followed by Holm-Sidak's post-hoc. Statistical comparisons between two groups were performed using two-tailed *Student's* unpaired t-tests. All data presented as mean $\pm$ SEM. & denotes effect of DiD, # denotes interaction (#p<0.05, ##p<0.01), $ denotes effect of virus ($p<0.05, $$p<0.01), * denotes post-hoc p<0.05, ** denotes post-hoc p<0.01. Source data are provided as a Source Data file.

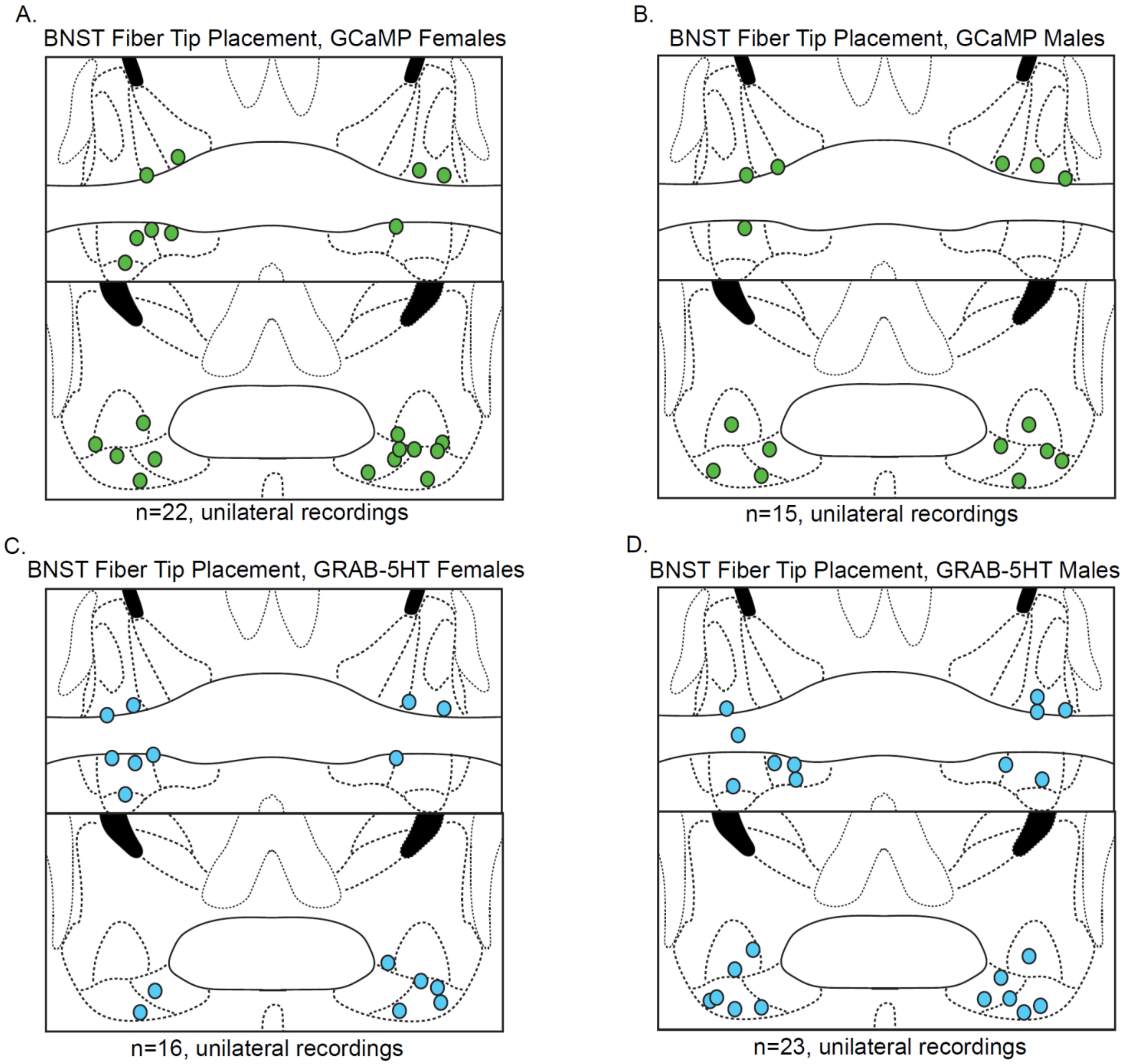

Figure S13: BNST fiber tip placements for GCaMP and GRAB-5HT experiments. A, BNST GCaMP females. **B,** BNST GCaMP males. **C,** BNST GRAB-5HT females. **D,** BNST GRAB-5HT males.

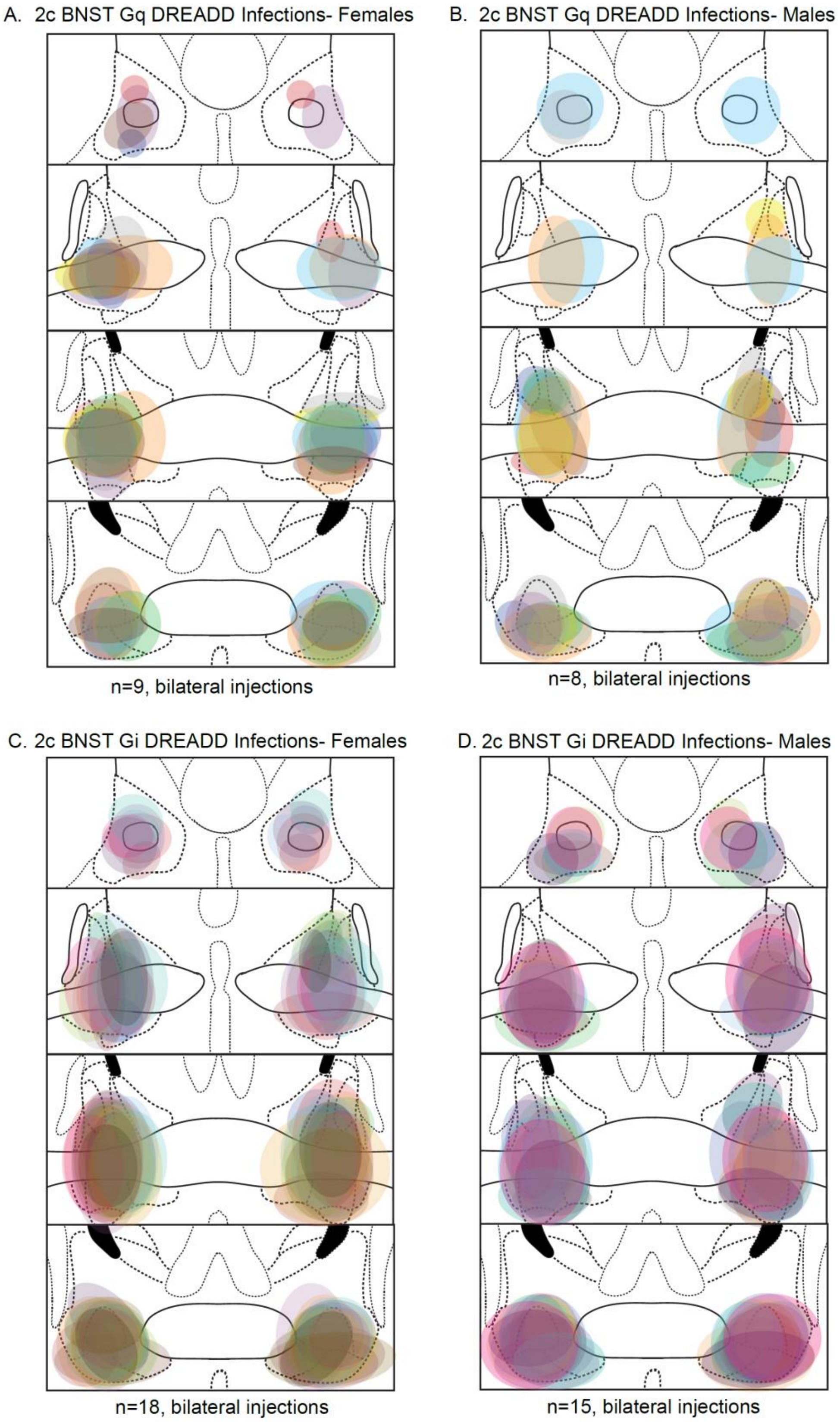

Figure S14: BNST DREADD viral spread (AAV-hSyn-DIO-hM3Dq or –hM4Di). A, BNST Gq females. **B,** BNST Gq males. **C,** BNST Gi females. **D,** BNST Gi males. Each unique color represents the viral spread for a single animal across the anterior-posterio axis of the BNST.

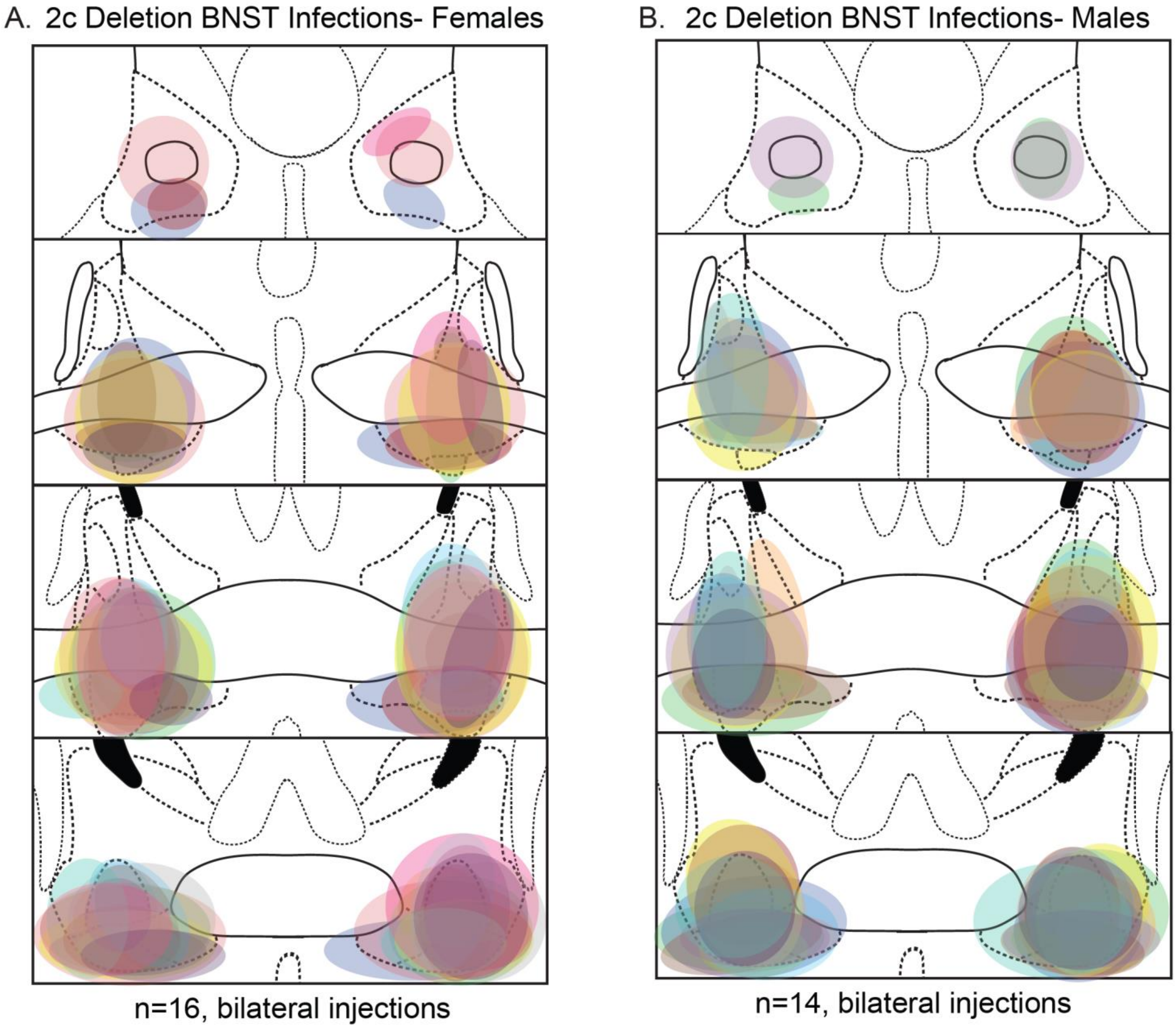

Figure S15: BNST $5HT_{2c}$ deletion viral spread (AAV-Cre-GFP). A, BNST females. **B,** BNST males. Each unique color represents viral spread for a single animal across the anterior-posterior axis of the BNST.

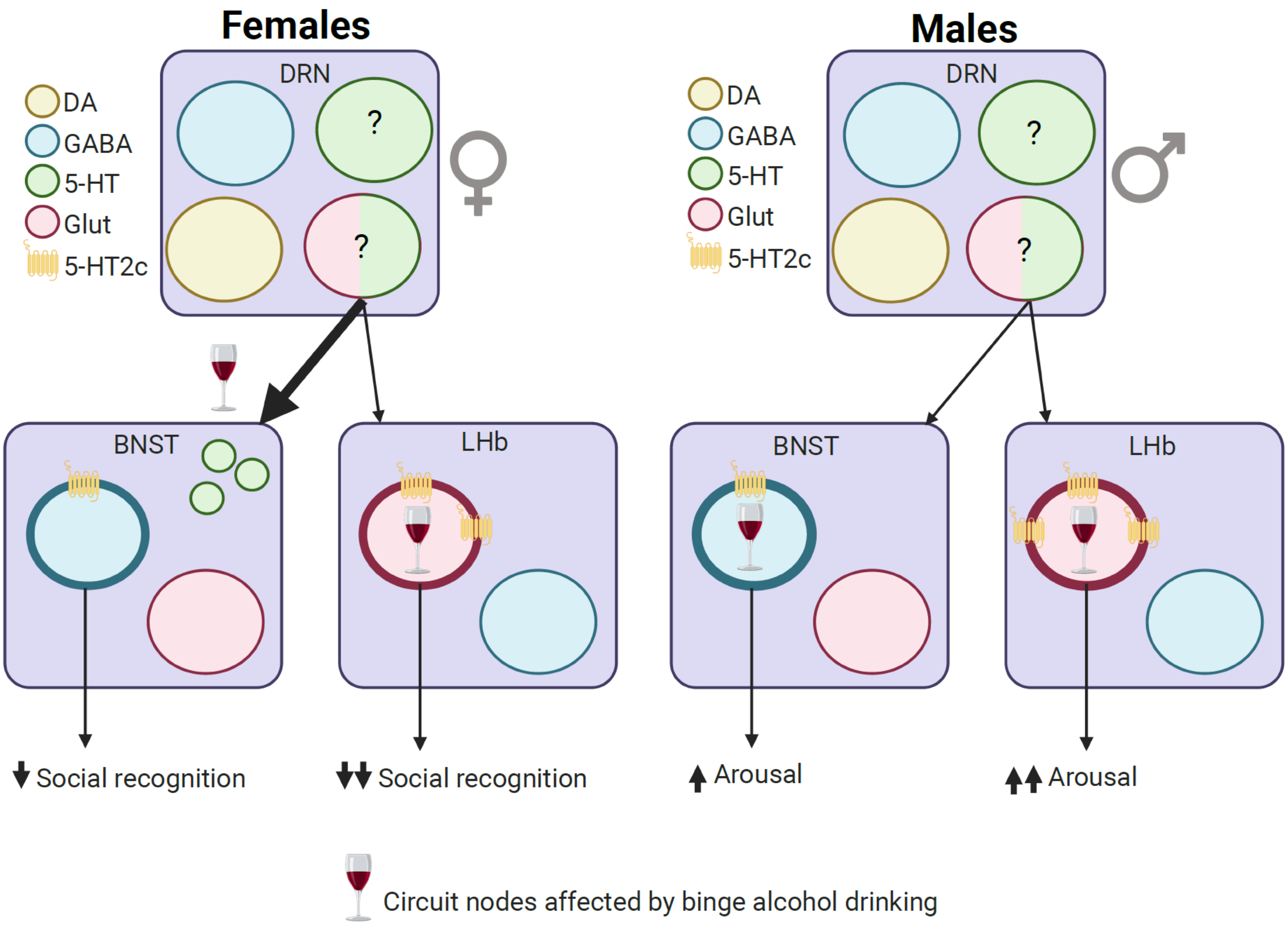

Figure S16: Summary of binge alcohol effects on DRN-BNST and DRN-LHb circuitry. Single 5-HT neurons in the DRN project to both BNST and LHb. In females, binge alcohol consumption increases 5-HT release in the BNST upon social interaction, decreases $BNST_{5HT2c}$ excitability in slice, and increases medial LHb_{5HT2c} excitability in slice. $5HT_{2c}$ in the BNST and the LHb partially regulate social recognition deficits induced by alcohol, but excessive activity of LHb_{5HT2c} predominantly drives alcohol-induced social recognition deficits in females. In males, binge alcohol consumption increases LHb_{5HT2c} and $BNST_{5HT2c}$ activity upon acoustic startle delivery, increases LHb_{5HT2c} excitability in slice, and increases $5HT_{2c}$ expression in the LHb. $5HT_{2c}$ in the BNST partially regulates arousal disturbances induced by alcohol, but excessive activity of LHb_{5HT2c} is the primary causal mechanism driving this effect. Created with Biorender.com.
