# Supplementary material for: Subcortical serotonin 5HT2c receptor-containing neurons sex-specifically regulate binge-like alcohol consumption, social, and arousal behaviors in mice
Source: Nat Commun. 2023 Mar 31;14:1800. doi: 10.1038/s41467-023-36808-2 (PMC10066391; doi:10.1038/s41467-023-36808-2)
Supplement: Supplementary file 3 — Reporting Summary [file 41467_2023_36808_MOESM3_ESM.pdf]

nature portfolio

Corresponding author(s): Thomas Kash

Last updated by author(s): Dec 29, 2022

Reporting Summary

Nature Portfolio wishes to improve the reproducibility of the work that we publish. This form provides structure for consistency and transparency in reporting. For further information on Nature Portfolio policies, see our Editorial Policies and the Editorial Policy Checklist.

Statistics

For all statistical analyses, confirm that the following items are present in the figure legend, table legend, main text, or Methods section.

n/a	Confirmed	
☐	☒	The exact sample size (*n*) for each experimental group/condition, given as a discrete number and unit of measurement
☐	☒	A statement on whether measurements were taken from distinct samples or whether the same sample was measured repeatedly
☐	☒	The statistical test(s) used AND whether they are one- or two-sided<br>*Only common tests should be described solely by name; describe more complex techniques in the Methods section.*
☒	☐	A description of all covariates tested
☐	☒	A description of any assumptions or corrections, such as tests of normality and adjustment for multiple comparisons
☐	☒	A full description of the statistical parameters including central tendency (e.g. means) or other basic estimates (e.g. regression coefficient) AND variation (e.g. standard deviation) or associated estimates of uncertainty (e.g. confidence intervals)
☐	☒	For null hypothesis testing, the test statistic (e.g. *F*, *t*, *r*) with confidence intervals, effect sizes, degrees of freedom and *P* value noted<br>*Give P values as exact values whenever suitable.*
☒	☐	For Bayesian analysis, information on the choice of priors and Markov chain Monte Carlo settings
☒	☐	For hierarchical and complex designs, identification of the appropriate level for tests and full reporting of outcomes
☒	☐	Estimates of effect sizes (e.g. Cohen's *d*, Pearson's *r*), indicating how they were calculated

Our web collection on statistics for biologists contains articles on many of the points above.

Software and code

Policy information about availability of computer code

Data collection	Ethovision XT 14.0 (Noldus), pClamp10.7, Bonsai 2.7.0 (Giancarlo Lopes, Open Source)
Data analysis	MATLAB R202a (Mathworks), Easy Electrophysiology 2.5 (Easy Electrophysiology, Open Source), Excel 2016 (Microsoft), GraphPad Prism 9 (GraphPad Software), Zen Blue 2.7 (Zeiss)

For manuscripts utilizing custom algorithms or software that are central to the research but not yet described in published literature, software must be made available to editors and reviewers. We strongly encourage code deposition in a community repository (e.g. GitHub). See the Nature Portfolio guidelines for submitting code & software for further information.

Data

Policy information about availability of data

All manuscripts must include a data availability statement. This statement should provide the following information, where applicable:

- Accession codes, unique identifiers, or web links for publicly available datasets
- A description of any restrictions on data availability
- For clinical datasets or third party data, please ensure that the statement adheres to our policy

Source data are provided with this paper as a Source Data file (Source Data Flanigan et al 2022). Raw Fiber Photometry data and associated videos are promptly available upon request, but are not immediately available to download due to file size. Please email the corresponding author Dr. Thomas Kash (Thomas_kash@med.unc.edu) to obtain these data.

March 2021

Human research participants

Policy information about studies involving human research participants and Sex and Gender in Research.

Reporting on sex and gender	*Use the terms sex (biological attribute) and gender (shaped by social and cultural circumstances) carefully in order to avoid confusing both terms. Indicate if findings apply to only one sex or gender; describe whether sex and gender were considered in study design whether sex and/or gender was determined based on self-reporting or assigned and methods used. Provide in the source data disaggregated sex and gender data where this information has been collected, and consent has been obtained for sharing of individual-level data; provide overall numbers in this Reporting Summary. Please state if this information has not been collected. Report sex- and gender-based analyses where performed, justify reasons for lack of sex- and gender-based analysis.*
Population characteristics	*Describe the covariate-relevant population characteristics of the human research participants (e.g. age, genotypic information, past and current diagnosis and treatment categories). If you filled out the behavioural & social sciences study design questions and have nothing to add here, write "See above."*
Recruitment	*Describe how participants were recruited. Outline any potential self-selection bias or other biases that may be present and how these are likely to impact results.*
Ethics oversight	*Identify the organization(s) that approved the study protocol.*

Note that full information on the approval of the study protocol must also be provided in the manuscript.

Field-specific reporting

Please select the one below that is the best fit for your research. If you are not sure, read the appropriate sections before making your selection.

☒ Life sciences ☐ Behavioural & social sciences ☐ Ecological, evolutionary & environmental sciences

For a reference copy of the document with all sections, see nature.com/documents/nr-reporting-summary-flat.pdf

Life sciences study design

All studies must disclose on these points even when the disclosure is negative.

Sample size	Sample size was determined by power calculations with alpha=0.05, power=0.8, and effect sizes estimated from previous experiments (~0.5-1.0)
Data exclusions	Data points were excluded for the following reasons: missed targeting of viral injections or optic fiber placements, clogged or spilled water/sucrose/alcohol bottles, malfunctions in fiber photometry hardware affecting quality of data collected, cells in electrophysiology experiments that did not meet inclusion criteria for health (see electrophysiology methods section). For behavioral data, individual mouse data points deemed statistically significant outliers were removed only if they altered the statistical conclusions of the experiment. Statistically significant outliers were identified using Grubb's test for outliers. This test was performed only once per dataset to find a maximum of one outlier data point.
Replication	All attempts at replication were successful. DREADD and 5HT2c deletion studies were performed independently in two different cohorts. The finding that binge alcohol consumption disrupts social recognition in females was reproduced in at least three independent cohorts.
Randomization	Group allocation was random.
Blinding	Investigators were not blind to group allocation for data collection but were blind to group allocation for data analysis. Blinding was not feasible for data collection as the same lead investigator performed surgeries, alcohol drinking experiments, and social/arousal behavior experiments.

Reporting for specific materials, systems and methods

We require information from authors about some types of materials, experimental systems and methods used in many studies. Here, indicate whether each material, system or method listed is relevant to your study. If you are not sure if a list item applies to your research, read the appropriate section before selecting a response.

Materials & experimental systems

n/a	Involved in the study
☐	☒ Antibodies
☒	☐ Eukaryotic cell lines
☒	☐ Palaeontology and archaeology
☐	☒ Animals and other organisms
☒	☐ Clinical data
☒	☐ Dual use research of concern

Methods

n/a	Involved in the study
☒	☐ ChIP-seq
☒	☐ Flow cytometry
☒	☐ MRI-based neuroimaging

Antibodies

Antibodies used	1. Rabbit polyclonal anti c-Fos (Synaptic Systems Cat# 226-003, RRID AB_2231974)<br>2. Mouse monoclonal anti-RFP (Rockland Antibodies and Assays, Cat#200-301-379, RRID AB_2611063)<br>3. Goat polyclonal anti-5HT (Immunostar, Cat#20079, RRID: AB_572262)<br>4. Donkey anti-mouse Cy2 (Jackson Immunoresearch, Cat#715-125-150)<br>5. Donkey anti-goat Cy3 (Jackson immunoresearch, Cat#705-165-003)<br>6. Donkey anti-rabbit Cy2 (Jackson Immunoresearch, Cat#711-225-152)
Validation	Rabbit polyclonal anti-c-Fos 226-003 from Synaptic Systems has been discontinued and is now 226-008. The supplier verifies 226-003 is specific for c-Fos but does not provide data (https://www.sysy.com/product/226008). We verified that the anti-RFP primary antibody does not react to wild-type tissue. According to the Immunostar website, the anti-5HT antibody was quality control tested using standard immunohistochemical methods (https://www.immunostar.com/product/5-ht-serotonin-rabbit-antibody/). We also did not observe 5HT+ cell bodies outside of the expected brain regions (DRN, MRN, etc).

Animals and other research organisms

Policy information about studies involving animals; ARRIVE guidelines recommended for reporting animal research, and Sex and Gender in Research

Laboratory animals	Adult (>8 weeks) male and female mice of the following strains were used as experimental mice in this study:<br>1. C57BL6/J wild-type (Jackson, Stock#000664)<br>2. 5HT2c-cre (bred in house, provided by Dr. Laura Heisler, Burke et al. 2013)<br>3. 5HT2c-lox/lox (bred in house, provided by Dr. Joel Elmquist, Berglund et al. 2013)<br>4. 5HT2c-Ai9 (Ai9 x 5HT2c-cre, bred in house, Ai9: Jackson Stock#007909.<br><br>All transgenic mice bred in house were on a C57BL6/J genetic background.<br><br>Adolescent (5-6 weeks) male and female albino C57BL6/J mice (B6(Cg)-TryC-2JI) were used as social target mice (Jackson stock#000058).<br><br>All mice were housed in polycarbonate cages (GM500, Techniplast) under a 12:12h reverse dark-light cycle where lights turned off at 7:00 am. Housing rooms were temperature (70-75°F) and humidity (40-60%) controlled. Mice had ad-libitum access to food (Prolab Isopro RMH 3000, LabDiet) and water unless otherwise stated. Experimental mice were group housed for all experiments until the beginning of Drinking in the Dark (DiD), at which point they were single housed until experiment completion. Social target mice were group housed.
Wild animals	Wild animals were not used in this study.
Reporting on sex	Findings in this paper apply to both sexes, as all experiments were performed separately in groups of males and females. Thus, male and female effects were analyzed independently but side-by-side.
Field-collected samples	There were no field collected samples in this study.
Ethics oversight	All experiments were approved by the UNC School of Medicine Institutional Animal Care and Use Committee (IACUC) and are in accordance with the NIH guidelines for the care and use of laboratory animals.

Note that full information on the approval of the study protocol must also be provided in the manuscript.
